# DynaSim: A MATLAB Toolbox for Neural Modeling and Simulation

**DOI:** 10.3389/fninf.2018.00010

**Published:** 2018-03-15

**Authors:** Jason S. Sherfey, Austin E. Soplata, Salva Ardid, Erik A. Roberts, David A. Stanley, Benjamin R. Pittman-Polletta, Nancy J. Kopell

**Affiliations:** ^1^Department of Mathematics and Statistics, Boston University, Boston, MA, United States; ^2^Center for Systems Neuroscience, Psychological and Brain Sciences, Boston University, Boston, MA, United States; ^3^Graduate Program for Neuroscience, Boston University, Boston, MA, United States; ^4^Department of Biomedical Engineering, Boston University, Boston, MA, United States

**Keywords:** dynamical systems, neural models, GNU octave, neuroscience gateway, graphical user interface, code generation, code:matlab

## Abstract

DynaSim is an open-source MATLAB/GNU Octave toolbox for rapid prototyping of neural models and batch simulation management. It is designed to speed up and simplify the process of generating, sharing, and exploring network models of neurons with one or more compartments. Models can be specified by equations directly (similar to XPP or the Brian simulator) or by lists of predefined or custom model components. The higher-level specification supports arbitrarily complex population models and networks of interconnected populations. DynaSim also includes a large set of features that simplify exploring model dynamics over parameter spaces, running simulations in parallel using both multicore processors and high-performance computer clusters, and analyzing and plotting large numbers of simulated data sets in parallel. It also includes a graphical user interface (DynaSim GUI) that supports full functionality without requiring user programming. The software has been implemented in MATLAB to enable advanced neural modeling using MATLAB, given its popularity and a growing interest in modeling neural systems. The design of DynaSim incorporates a novel schema for model specification to facilitate future interoperability with other specifications (e.g., NeuroML, SBML), simulators (e.g., NEURON, Brian, NEST), and web-based applications (e.g., Geppetto) outside MATLAB. DynaSim is freely available at http://dynasimtoolbox.org. This tool promises to reduce barriers for investigating dynamics in large neural models, facilitate collaborative modeling, and complement other tools being developed in the neuroinformatics community.

## 1. Introduction

**Figure 1:**
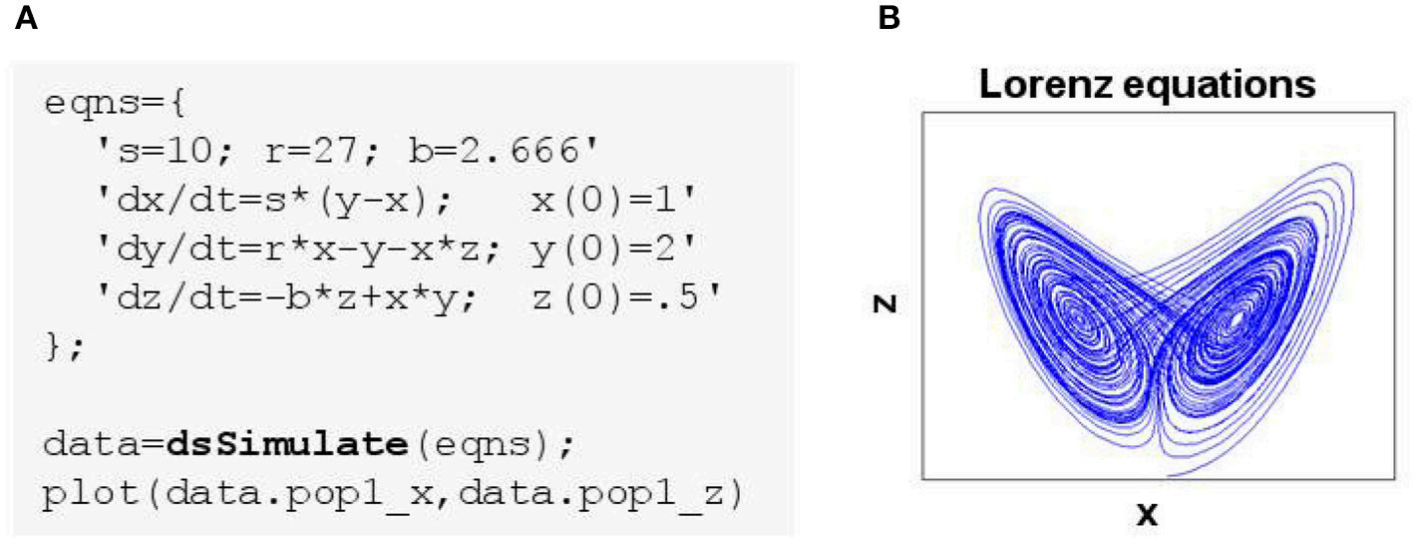
Simulating a simple system of ordinary differential equations in DynaSim. **(A)** MATLAB code using the DynaSim toolbox. Simulation is achieved by passing a model specification to the DynaSim dsSimulate function. Simulated data are returned in a DynaSim data structure. **(B)** (x,z) phase plane of Lorenz system.

**Figure 2:**
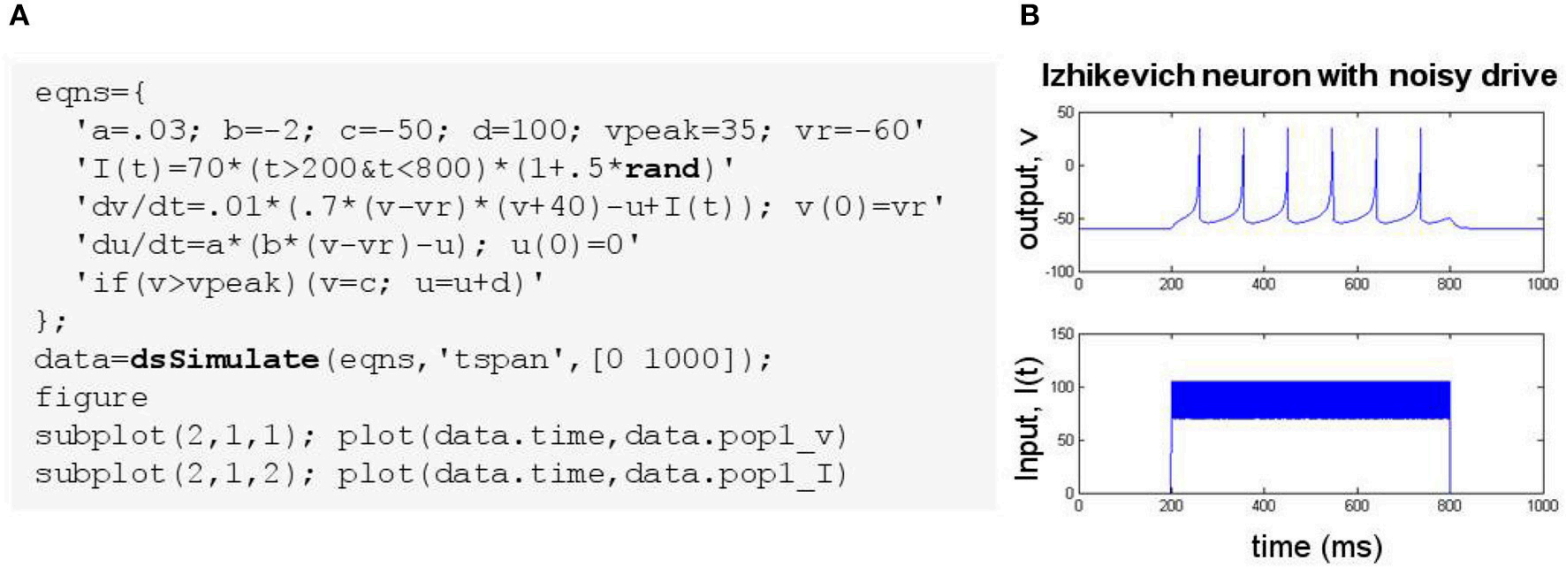
Simulating an ODE system with conditional reset and stochastic drive. **(A)** MATLAB code using the DynaSim toolbox. The model is specified using a cell array of strings, eqns, listing equations defining parameters, an input function I(t), ODEs with ICs, and a conditional reset. The stochastic input uses the built-in MATLAB function rand. **(B)** Plot of the time-varying input and simulated output.

**Figure 3:**
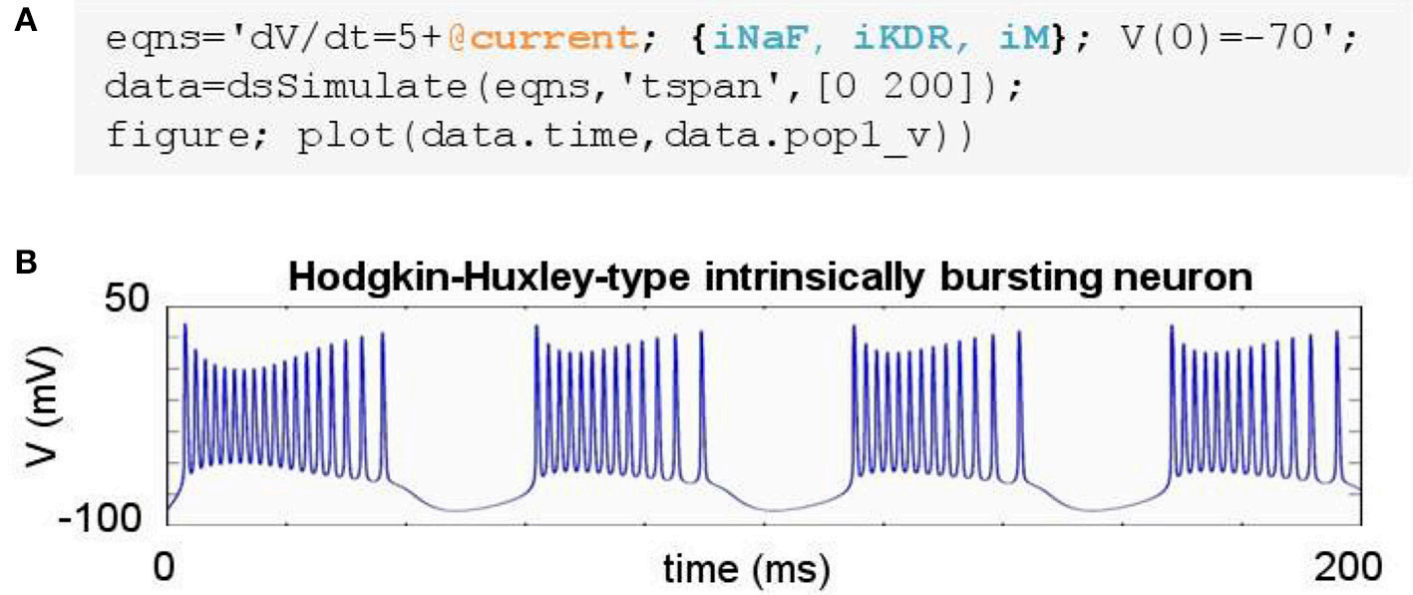
Simulating a biophysically-detailed neuron model using mechanisms. **(A)** DynaSim model leveraging existing model objects for iNaF, iKDR, and iM currents to simplify the specification of a detailed neuron model. **(B)** IB response to tonic current.

**Figure 4:**
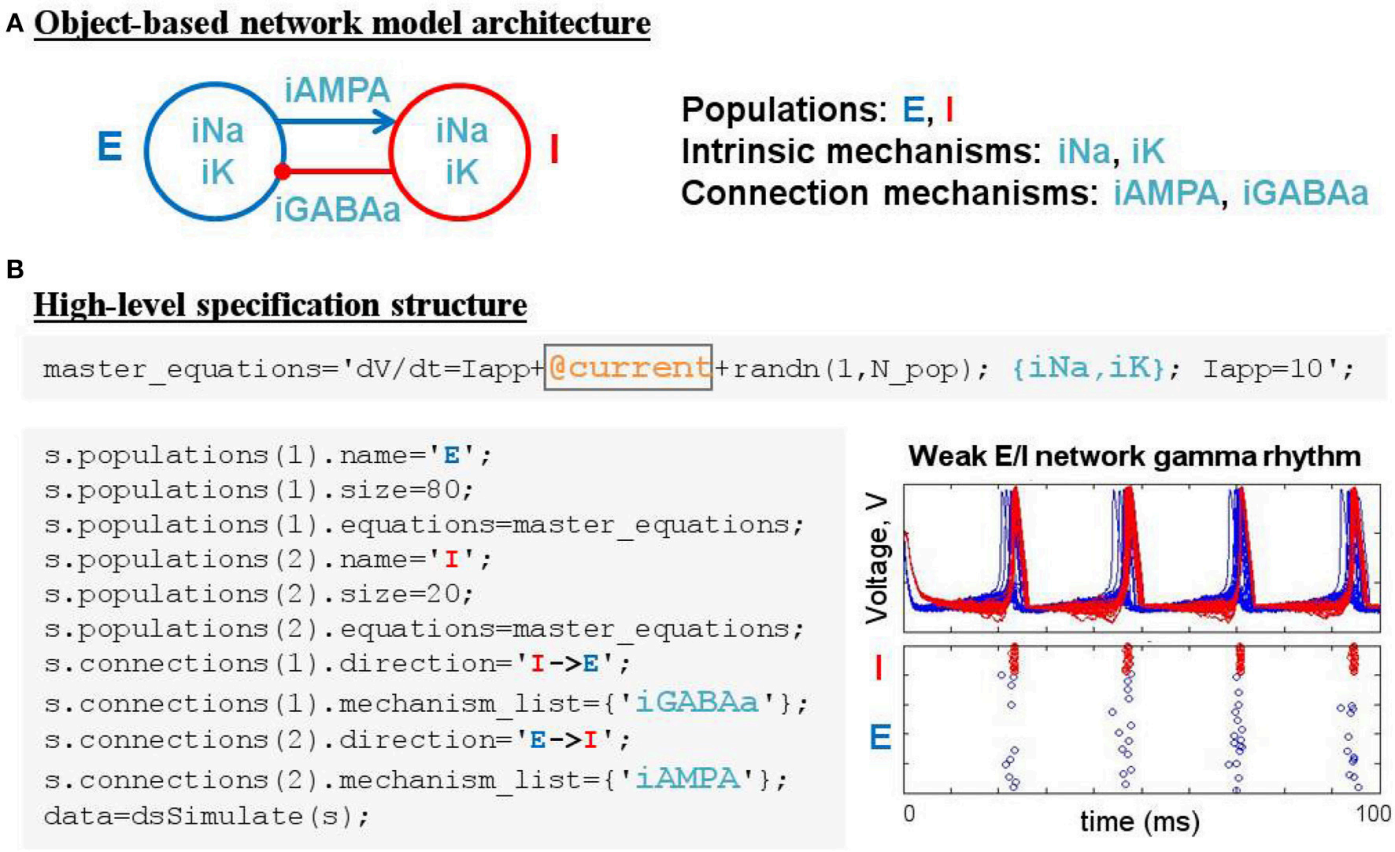
Simulating weak PING rhythms using a model specification structure. **(A)** The conceptual object-based architecture of a biophysically-detailed network of excitatory (blue) and inhibitory (red) cells. **(B)** Mapping the object-based architecture onto a DynaSim specification structure that contains all the high-level information necessary to construct the complete system of equations for the full model using objects from a library of pre-existing ionic mechanisms.

DynaSim (http://dynasimtoolbox.org) is a MATLAB (MATLAB, 2017) and GNU Octave (Eaton et al., 2016) toolbox developed for rapid prototyping of large neural models and batch simulation management. It enables researchers to focus on model details instead of implementation, while making it easy to share and explore models with the rest of the community. It facilitates rapid prototyping of neural models by enabling networks of neurons with one or more compartments to be specified by any combination of: (1) equations with conventional mathematical notation (Figures 1, 2), similar to XPP (Ermentrout, 2002) and the Brian simulator (Goodman and Brette, 2008), (2) built-in MATLAB functions, and (3) predefined, mechanistically-meaningful model objects (Figures 3, 4), similar to objects in Brian, mechanisms in NEURON (Hines and Carnevale, 1997), and nodes/connections in NEST (Gewaltig and Diesmann, 2007). DynaSim's higher-level specification, described below, easily scales to arbitrarily complex population models and networks of interconnected populations (Figure 4), and does not require significant “boilerplate” code for even very large networks.

In addition to neural modeling, it provides a simple, general-purpose interface for numerically integrating all models supported by MATLAB's built-in solvers for ordinary differential equations. Its compatibility with GNU Octave enables it to be used for free by those without a MATLAB license. It can also be used for free through a web browser using the Neuroscience Gateway web portal (https://www.nsgportal.org). DynaSim is most similar to the Brian simulator in spirit, scope, and its ability to simulate models based on equations as well as libraries of pre-existing model objects (Goodman and Brette, 2009). However, DynaSim provides both script-based and easy-to-use graphical interfaces as well as better support for analyzing and exploring model dynamics over parameter space than other neural simulators. The software has been implemented in MATLAB because MATLAB lacks advanced tools for neural modeling, despite its popularity, especially among neuroscientists, and a growing interest in modeling neural systems. DynaSim incorporates the best features of existing simulators to fill this niche in MATLAB, and it leverages MATLAB's extensive capabilities to provide features that are lacking in other simulators.

**Figure 5:**
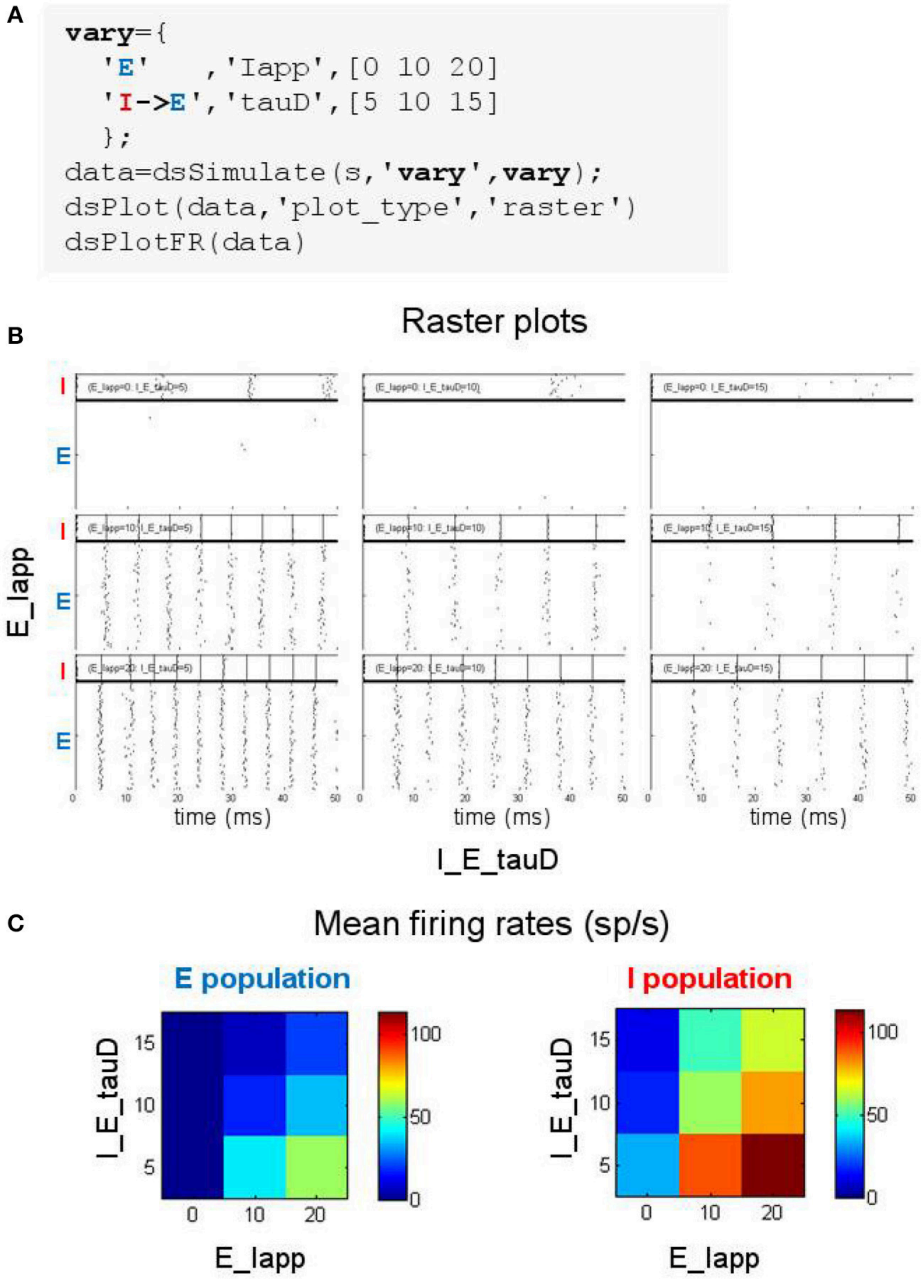
Searching parameter space using the DynaSim toolbox. **(A)** MATLAB code using the DynaSim dsSimulate function with the vary option to specify a set of 9 simulations varying two parameters (Iapp in population E and tauD of the connection from I to E). **(B)** Raster plots produced by dsPlot with the plot_type option given an array of DynaSim data structures containing results for all 9 simulations. **(C)** Plots produced by dsPlotFR showing how mean firing rates for E and I populations change as a function of the two varied parameters.

**Figure 6:**
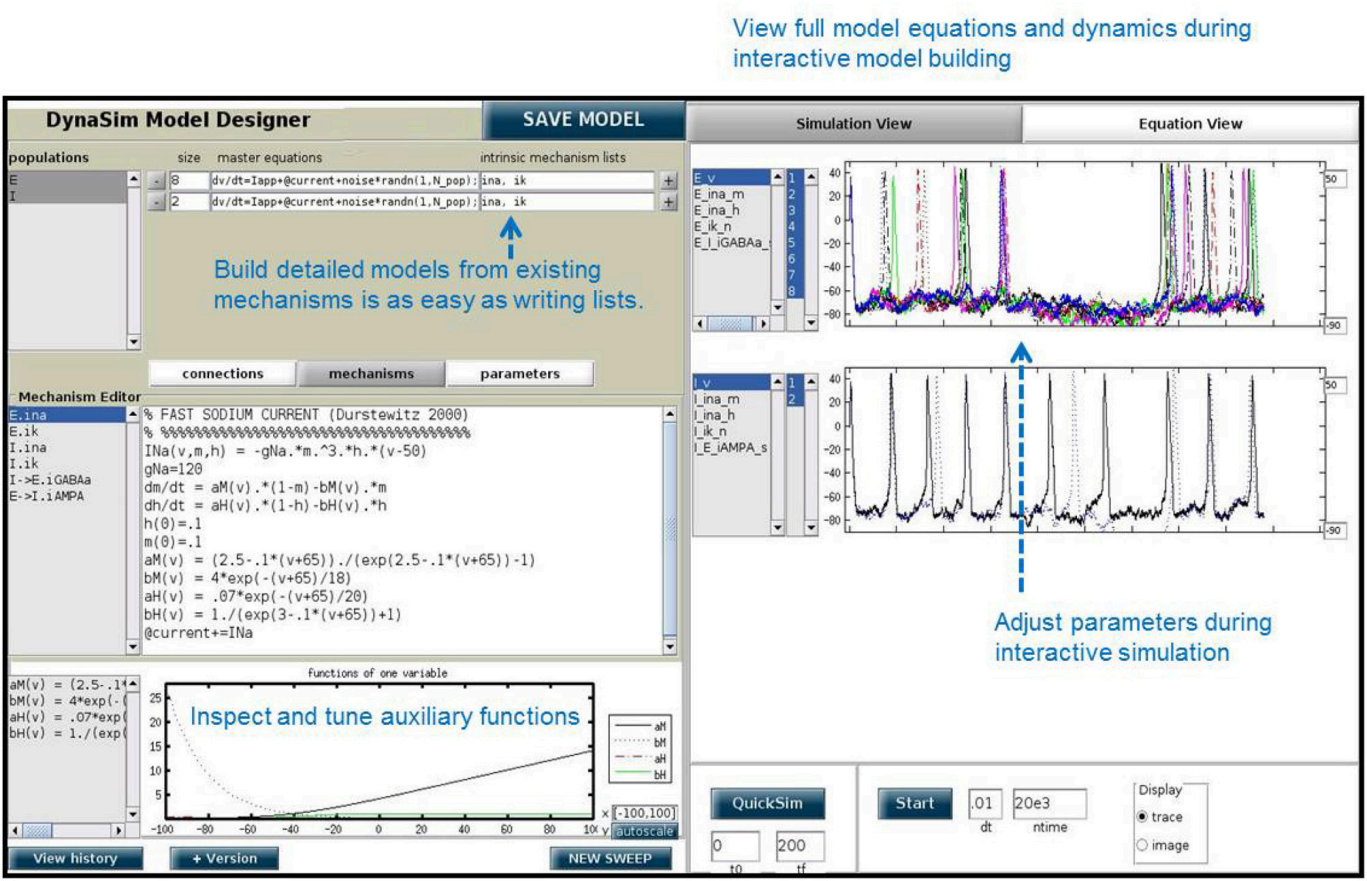
DynaSim Graphical User Interface showing the weak PING model.

DynaSim includes a large set of features to simplify the processes of exploring model dynamics over parameter spaces (Figure 5), running separate simulations in parallel on multicore processors and computer clusters, as well as parallel analysis and plotting of large numbers of simulated data sets. It increases simulation speed, compared to common MATLAB implementations, using a combination of optimized vector computation, C compilation, and parallel simulation. It includes a graphical user interface (DynaSim GUI) that supports full functionality without requiring user programming (Figure 6). The GUI is a useful aid for teaching about the dynamics of neural systems and is more accessible to experimentalists and students without a background in mathematics and programming. The design of DynaSim incorporates a novel schema for model specification to facilitate interoperability with other tools outside MATLAB including simulator-independent specifications (e.g., NeuroML, Gleeson et al., 2010, SBML, Hucka et al., 2003), simulators (e.g., NEURON, Hines and Carnevale, 1997, Brian, Goodman and Brette, 2008, NEST, Gewaltig and Diesmann, 2007), model repositories (e.g., Open Source Brain, http://www.opensourcebrain.org), and web-based applications (e.g., Geppetto, http://www.geppetto.org). This tool aims to simplify the investigation of dynamics of complex neural network models, facilitate collaborative modeling, and complement other tools being developed in the neuroinformatics community.

This paper begins with Worked_Examples demonstrating the simplicity and power of DynaSim for rapid prototyping and model exploration. Next, DynaSim's Technical Details will be described, followed by a comparison to other simulators. The paper concludes with a discussion of future developments.

## 2. Worked examples

In DynaSim, users pass a model specification to the function dsSimulate, which integrates the model equations and returns a data structure containing the simulated data. Models can be specified using strings or a MATLAB structure, and large models can be specified easily from combinations of existing model components. The following examples will demonstrate the specification of increasingly complex models and advanced DynaSim capabilities provided by optional arguments in dsSimulate.

### 2.1. Example 1: lorenz equations

Any system of ordinary differential equations (ODEs) can be modeled in DynaSim by listing equations using conventional mathematical notation. Equations can be listed in a single string or a cell array of strings and may contain parameters, functions, conditional statements, ODEs and their initial conditions (ICs). To demonstrate the generality of this approach, the Lorenz equations (Lorenz, 1963):

(1) $$\begin{array}{l} \frac{dx}{dt}=s(y-x)\\ \frac{dy}{dt}=rx-y-xz\\ \frac{dz}{dt}=-bz+xy \end{array}$$

are defined in the cell array eqns in Figure 1A, and the system is numerically integrated by passing this user specification (i.e., eqns) to the DynaSim function dsSimulate. Integration in DynaSim is described in the Technical Details. The results are plotted in Figure 1B. The same approach can be applied to simulate ODE-based rate models of neural systems.

### 2.2. Example 2: izhikevich spiking neuron model

The Izhikevich neuron (Izhikevich, 2003) is a system of differential equations with a conditional update:

(2) $$\begin{array}{l} \frac{dv}{dt}=.01(.7(v-v_{r})(v+40)-u+I(t))\\ \frac{du}{dt}=a(b(v-v_{r})-u)\\ \text{if }v>v_{peak},\text{then }v=c,u=u+d. \end{array}$$

Figure 2A demonstrates the specification of an Izhikevich model using a cell array of strings and a noisy time-varying input function that leverages the built-in MATLAB function rand. Input and simulated output are plotted in Figure 2B. Conditional updates are incorporated using the notation: if(condition)(actions), where condition is a MATLAB expression that evaluates to true or false and actions is a semicolon-delimited list of statements to execute when condition is true. Conditionals are evaluated on every time step after the model state is updated according to the differential equations. They can be used to update state variables or parameters when their prescribed conditions are satisfied.

### 2.3. Example 3: hodgkin-huxley-type spiking neuron models

The construction of large models with many equations can be greatly simplified by utilizing components from a library of pre-existing model objects. For instance, conductance-based neuron models often include component ion currents (i.e., ionic mechanisms) that may be used in models of different neuron types. A regular spiking (RS) neuron includes fast spike-generating sodium (e.g., iNaF) and potassium (e.g., iKDR) currents, while an intrinsically bursting (IB) neuron possesses the same spike-generating currents plus a slower potassium current (e.g., iM) providing a second time scale separating bursts of spikes. Both models rely on the same iNaF and iKDR currents while the IB model incorporates an additional iM current.

DynaSim expedites the construction of such models by leveraging pre-existing model objects (e.g., iNaF,iKDR,iM). Models incorporate reusable objects by including in their equations: (1) placeholders with an “@” sign for terms that rely on external model objects; and (2) a list of the external model objects (e.g., ionic mechanisms) defining the terms to be inserted wherever the appropriate placeholders appear. Figure 3A demonstrates the specification of a biophysically-detailed IB neuron using a placeholder, @current, in the voltage dynamics, dV/dt, and a list of ionic mechanisms, {iNaF,iKDR,iM}, defining currents that affect the voltage dynamics. Figure 3B plots the simulated response to a tonic injected current. The IB neuron could be rapidly converted into a RS neuron simply by reducing the list of mechanisms to {iNaF,iKDR}. Alternatively, the neuron model could be made arbitrarily more complex by adding as many ion currents as desired to the mechanism list. DynaSim comes pre-packaged with a variety of commonly used model objects (see Table 1 for a representative list). This feature is described further in the Technical Details section below.

**Table 1:** Representative list of model objects packaged with the default library.

**Object**	**Description**
**Input mechanisms**	
poisson	Non-homogeneous poisson process
noise	Source of gaussian noise
stim	Source of tonic stimulation
**Intrinsic mechanisms**	
iNa	Fast sodium current (Hodgkin and Huxley, 1952)
iK	Fast potassium current (Hodgkin and Huxley, 1952)
iM	Slow, M-type potassium current (Traub et al., 2003)
iCaT	T-type calcium current (Ching et al., 2010)
iKCa	Calcium-dependent potassium current (Durstewitz et al., 2000)
iNaP	Persistent sodium current (Durstewitz et al., 2000)
iH	Hyperpolarization-activated cation h current (Ching et al., 2010)
CaBuffer	Calcium buffer (Durstewitz)
**Connection mechanisms**	
iAMPA	AMPA synapse: sigmoid threshold with excitatory parameters (Kopell et al., 2000)
iGABAa	GABA_A_ synapse: sigmoid threshold with inhibitory parameters (Kopell et al., 2000)
iGABAb	GABA_B_ synapse (Ching et al., 2010)
iNMDA	NMDA synapse (Koch and Segev, 1998)
iGAP	Ohmic gap junction
iCOM	Axial current for connecting two compartments
**Populations**	
LIF	Leaky integrate-and-fire neurons
Izh	Izhikevich neurons (Izhikevich, 2003)
ML	Morris-Lecar neurons (Morris and Lecar, 1981)
FHN	FitzHugh-Nagumo neurons (FitzHugh, 1955)
HH	Hodgkin-Huxley neurons with iNa and iK currents (Hodgkin and Huxley, 1952)
RS	Cortical Regular Spiking neurons (Kramer et al., 2008)
IB	Cortical Intrinsically Bursting neurons (Kramer et al., 2008)
FS	Cortical Fast Spiking interneurons (Kramer et al., 2008)
LTS	Cortical Low Threshold Spiking interneurons (Kramer et al., 2008)

### 2.4. Example 4: weak PING spiking network model

The construction of large network models (see the Benchmarking section for limits on network size) is greatly simplified by introducing an additional model object: the population. Connections between populations and dynamics of population constituents depend on lower-level mechanisms. Figure 4A demonstrates an object-based network architecture with two populations, named E and I, each with dynamics determined by ionic mechanisms. Voltage dynamics of the E population are shaped by intrinsic ion currents, named ina and ik, and an inhibitory synaptic current, named iGABAa, that depends on the state of the presynaptic I population. Similarly, the I population has voltage dynamics shaped by the same intrinsic currents (ina, ik) and an excitatory synaptic current (iAMPA) that depends on the E population. Due to the kinetics of the predefined ionic mechanisms and the parameters used in this example, the network generates a weak pyramidal-interneuron network gamma (PING) rhythm (Börgers and Kopell, 2005).

DynaSim expedites the process of specifying object-based network models using a DynaSim specification structure that organizes information about the population-level equations and the mechanisms on which they depend. To facilitate the computational implementation of an object-based conceptual network model (Figure 4A), information is organized into two fields of the specification structure: populations and connections. Each population has its own equations subfield, which can link to external model objects, as well as subfields specifying the name and size of the population. When populations have more than a single neuron, initial conditions (e.g., initial voltages) and model parameters (e.g., maximal synaptic conductances) can be made heterogeneous across the population by setting their values using arrays with one element per neuron. Connections between populations are made by connection mechanisms (e.g., synaptic currents) specified in the connections field. See the Technical Details section below for further information on the specification structure. Tutorials on using the specification to construct and combine large networks of multicompartment neurons as well as examples demonstrating the construction of large cortical and thalamic models can be found in the online documentation.

Patterns of connectivity between source and target populations are specified using connectivity matrices that appear in the equations of their connection mechanisms. Optionally, connectivity matrices can be defined in an external function or the same MATLAB script as the specification structure and stored as a parameter for the appropriate mechanism (see the dsDemos script included with DynaSim and the online “Getting started” tutorial for examples). Figure 4B demonstrates the DynaSim specification of the weak PING model shown in Figure 4A, as well as raster plots and an overlay of voltage traces showing a 40 Hz network oscillation in response to a tonic drive. This example uses the default all-to-all connectivity of the iAMPA and iGABAa mechanisms in the default DynaSim library. An extended example specifying custom connectivity in the PING model can be found online in the “Getting started” tutorial. Connectivity matrices can be made sparse by setting sparse_flag to 1 in the call to dsSimulate. Similar to the mechanism-based specification of the IB neuron in Example 3, the network model can be rapidly adjusted and made as complex as desired by simply updating mechanism lists for each population and for the connections between populations. Additionally, a configurable buffer of spike times, determined by upward threshold crossings, can be accessed using the reserved variables tspike_pre and tspike_post, for computing spike-timing dependent functions (e.g., for spike-timing-dependent plasticity, STDP); see the examples in the DynaSim toolbox and the online documentation for more information. It is worth noting that any network model specified using a specification structure can be equivalently specified using the method of explicitly listing all equations as described in Examples 1–2. However, this is least preferred, since it is more tedious, time-consuming, and error-prone than using predefined model objects.

### 2.5. Example 5: script-based modeling without writing equations

Predefined populations can be combined and remixed with predefined mechanisms using the specification structure to construct neural models without writing any equations. This approach enables experimentalists and students without mathematical proficiency to easily explore neural dynamics and dependence on various stimulation protocols and biophysical details. For instance, a population of 100 noise-driven neurons with Hodgkin-Huxley (HH) kinetics can be specified and simulated using the following script:


% Specify predefined HH neuron model
s.populations(1).equations=’HH’;
s.populations(1).size=100;
% Add Gaussian noise
s.populations(1).mechanism_list={’noise’};
% Specify noise amplitude
s.populations(1).parameters
      ={’noise_amp’,1e3};
% Run simulation
data = dsSimulate(s);


See Table 1 for a list of predefined neuron models that can be used without requiring the user to enter any equations. The example population can be converted into a model of leaky integrate-and-fire neurons, or any other population in Table 1, by simply changing the predefined neuron model specified in the equations field. See the online documentation for examples swapping out predefined models, including neurons with parameterized refractory periods. Furthermore, new mechanisms can be added to predefined neuron models by simply adding them to the mechanism list. The specification can be expanded to include any number of additional populations and network-forming connections between them (see Example 4).

Thus, DynaSim offers a range of model specification methods from writing detailed equations (for computational scientists, engineers, and mathematicians) to simply listing objects from the model library, enabling specification of complex models without writing equations (for experimentalists and students).

### 2.6. Example 6: exploring parameter space of the weak PING model

One of the strengths of DynaSim is its support for exploring how system behavior changes as a model is systematically varied. In the simplest case, exploration involves simulating a model with varying sets of parameters followed by analysis and visualization of the results over the parameter space. This can easily be performed in DynaSim by setting the vary option of dsSimulate using a compact specification of the parameter space to explore. For instance, the space can be specified using a set of triplets (as in Figure 5A) with each element indicating the values to use for parameters of populations and/or connections; the space to explore is then constructed from the Cartesian product of the parameter values from the set of triplets. Aside from Cartesian products, DynaSim offers multiple forms of specification to accommodate different patterns in parameter space.

Exploring the weak PING model is demonstrated in Figure 5A where 9 simulations are specified with three values for each of two parameters: the amplitude of the current injected into cells of the E population (Iapp) and the inhibition time constant of the inhibitory synapse onto E cells (tauD). DynaSim provides multiple functions for visualizing results over parameter space. For instance, Figure 5B shows raster plots produced by the DynaSim dsPlot function called in Figure 5A, while Figure 5C, produced by the DynaSim dsPlotFR function, shows the dependence of average firing rates on varied parameters.

The DynaSim dsSimulate function offers three important options for increasing the speed of simulation. The benchmarks described below show that the speed of most simulations can be increased by up to a factor of 10x by setting the compile_flag option to 1:


data = dsSimulate(s,’vary’,vary,
                    ’compile_flag’,1);


which directs dsSimulate to compile the simulation into C code (i.e., MATLAB MEX compilation) before numerical integration. Furthermore, the time required to run a set of simulations can be decreased by running multiple simulations simultaneously in parallel either on the user's local machine or on a computer cluster. Simulations can be run in parallel on the user's machine using the parfor function from the MATLAB Parallel Computing Toolbox; this feature can be activated by setting the parfor_flag option to 1:


data = dsSimulate (s,’vary’,vary,
                     ’parfor_flag’,1);


Additionally, a cluster can be used to parallelize a set of simulations. Clusters have “login nodes” that users can access, and “compute nodes” that jobs access. Typically, users submit jobs to a batch queueing system from the login node, and the queueing system controls the execution of the code on compute nodes. As described in the Technical Details section, DynaSim automates the creation and submission of jobs that run simulations, perform analyses, and plot results on compute nodes of a cluster with the Sun Grid Engine queueing system. This feature can be activated on a login node by setting the cluster_flag option to 1, the study_dir option to a directory where jobs should save outputs, and then using the DynaSim dsImport function to load all simulated data:


D = pwd; % where to save data
dsSimulate(s,’vary’,vary,
             ’cluster_flag’,1,
             ’study_dir’,D);
data = dsImport(D);


All three options (compile_flag, parfor_flag, and cluster_flag) can be used in combination to achieve multiplicative benefits. With minimal setup, these capabilities can facilitate utilization of extremely powerful compute cluster resources for the user. These options are described further in the Technical Details section.

### 2.7. Example 7: batch analysis and visualization of simulated data

The DynaSim simulator returns simulated data that can be manipulated directly by the user using any built-in capabilities of MATLAB. Beyond that, DynaSim provides post-simulation hooks that enable the same analysis and/or plotting function to be applied serially or in parallel to all output data sets (e.g., from a parameter sweep). At present, DynaSim provides analysis functions for computing firing rates, power spectra, and coherence; it provides plotting functions for generating state variable traces, raster plots, and power plots. See the online documentation for details. DynaSim also supports custom analysis and plotting functions. Custom functions must take a DynaSim data structure as their first argument and return results in a structure; they may have any number of additional arguments:


function results = my_analysis(data)
     % do something
     % return output in structure ’results’
end


Analysis functions are specified as function handles using the analysis_functions option in dsSimulate, and plotting functions are specified similarly using the plot_functions option. For instance, simulations varying model parameters (see the vary option in Figure 5A) can be run and analyzed using the ‘my_analysis’ function in parallel on different nodes of a cluster by executing the following code from a login node (see Example 6 for details on cluster computing):


D = pwd; % where to save results
dsSimulate(s,’analysis_functions’,
      @my_analysis,’vary’,vary,
      ’cluster_flag’,1,’study_dir’,D);
results = dsImportResults(D,@my_analysis);


Analysis results are saved to study_dir and can be loaded using the dsImportResults function. For a single simulation, results will contain the output from ‘my_analysis.’ For a set of simulations, results is an array of structures with each element containing the output from ‘my_analysis’ for one simulation in the batch. Multiple analysis functions can be applied to each simulated data set by passing a cell array of function handles using the analysis_functions option. Post-simulation hooks work the same when simulations are run serially or in parallel on the local machine. These approaches can be used to apply a complex and possibly custom set of analyses to each simulated data set with options to store only analysis results (i.e., minimize disk space requirements) and to run analyses in parallel on different nodes of a compute cluster or different cores of a given machine.

Alternatively, multiple simulated data sets can be analyzed after all simulations are complete and data sets are loaded in memory using the dsAnalyze function:


results = dsAnalyze(data,@my_analysis);


This DynaSim function supports parallel processing using the parfor_flag option:


results = dsAnalyze(data,@my_analysis,
            ’parfor_flag’,1);


The combination of built-in support for common as well as custom analysis and visualization functions provided by DynaSim is designed to meet all needs of modelers and experimentalists seeking to explore model dynamics.

### 2.8. Example 8: exploring the weak PING model in DynaSim GUI

In addition to the DynaSim functions available for script-based model building and simulation, DynaSim provides a unique graphical user interface (DynaSim GUI) that enables users to access all of DynaSim's features without MATLAB programming. The DynaSim GUI provides a highly flexible and dynamic environment for interactive, real-time exploration of how model functions and dynamics vary with parameters, as well as how varying model architecture changes the system behavior. Any model can be explored using the GUI by passing its specification to the function dynasim. For instance, the GUI can be used to explore the weak PING model defined in Figure 4 by executing: dynasim(s). Figure 6 shows how the weak PING model appears in DynaSim GUI. Alternatively, the model could be built from scratch using the graphical interface.

A special feature of the DynaSim GUI is the ability to interactively modify a model during ongoing simulation and to observe the effects without needing to restart the simulation. This feature is useful for interactively exploring models and manually tuning model parameters. This feature is offered by few simulators.

The DynaSim GUI is especially useful as a teaching tool and for researchers without programming experience. Researchers who prefer writing code may still find it useful for prototyping and model exploration before choosing a model to investigate further in MATLAB scripts using functions of the DynaSim toolbox.

## 3. Technical details

### 3.1. Modeling

Models can be specified by the user with a cell array of strings (Examples 1–2), a single string (Example 3), or a specification structure (Examples 4–5), based on a combination of master equations (using standard mathematical notation and built-in MATLAB functions) and optional model objects from an existing library (Examples 3–5). This provides the user with multiple ways of specifying a model depending on the complexity of the model and the level of mathematical detail the user wishes to provide. Internally, DynaSim converts user-supplied information into a standardized high-level specification structure, which is subsequently converted into a lower-level model structure. The model structure is then used to automatically generate a suitable implementation (m-file, mex-file, or function handle) based on the desired simulation method. The results of simulation are returned in a DynaSim data structure. Simulation in DynaSim always involves sequential processing of the following DynaSim structures:


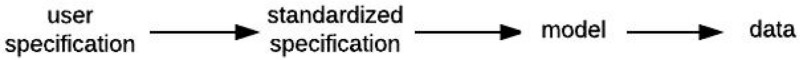


#### 3.1.1. Model objects for populations and mechanisms

Equations define parameters, variables, functions, and ODEs. Model objects are ways of grouping equations to facilitate the rapid construction of larger models. There are two types of objects: populations and mechanisms. Populations represent discrete systems of interest like populations of cells, individual cells, or compartments (e.g., soma, dendrite). Mechanisms represent smaller-scale components that affect the dynamics of populations (e.g., ion currents); they are called intrinsic mechanisms when they depend only on the state of the population they affect (e.g., sodium and potassium currents), and they are called connection mechanisms when they depend additionally on the state of other populations (e.g., synaptic currents). DynaSim comes prepackaged with a library of common model objects (see Table 1 for a representative list). Each object is assigned a unique name to enable the duplication of parameter, variable, and function names in different objects. The same intrinsic mechanism can be reused in different populations, and the same connection mechanism can be reused to connect different pairs of populations. Thus, mechanism objects enable equations to be specified once and reused an arbitrary number of times, and both types of objects enable equations to be specified without requiring the tedious assignment of unique variable/function names each time the same equations appear in a model.

#### 3.1.2. DynaSim structures for higher-level specification, lower-level model definition, and simulated data

**Figure 7:**
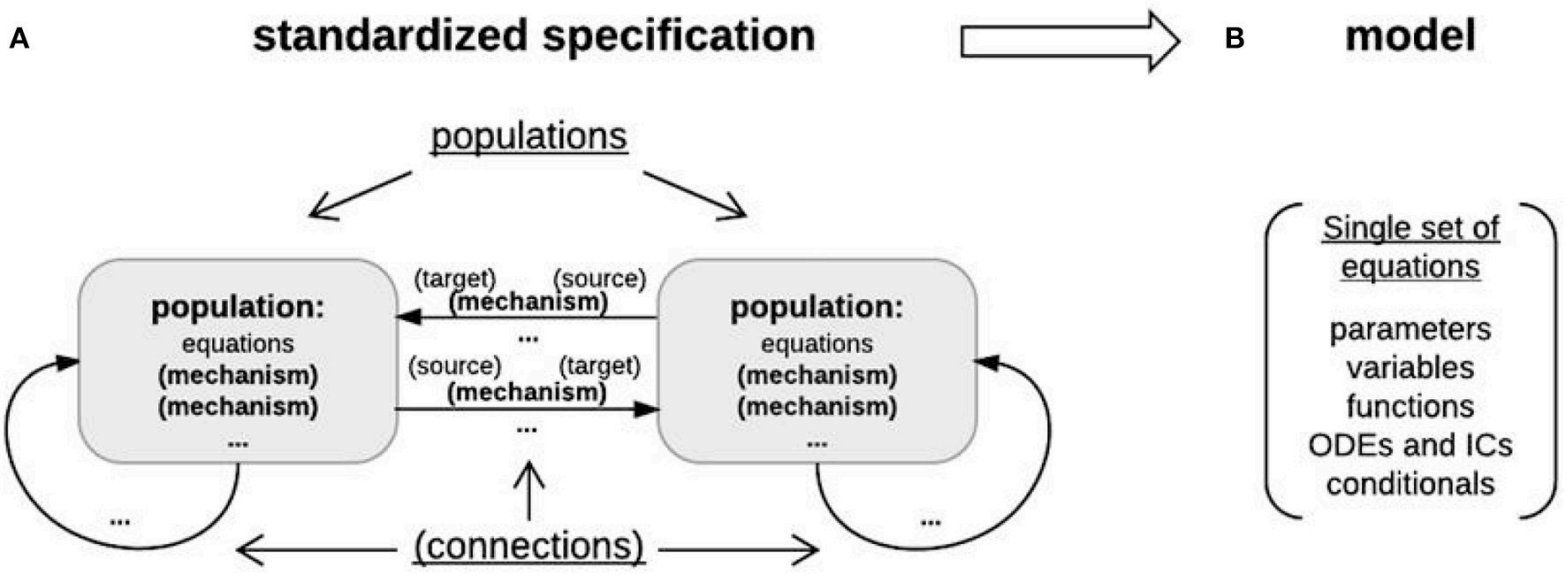
Object-based architecture, standardized specification, and DynaSim models. **(A)** Object-based architecture and standardized specification. Discrete model objects (populations and mechanisms) are shown in bold; any object can be stored independently in the library and reused as components of larger models. There is no limit on the number of objects in a DynaSim model. Fields of the standardized specification structure are underlined. Each population can have a list of intrinsic mechanisms; each directed pair of source and target populations can have a list of connection mechanisms. Optional objects are enclosed in parentheses. A string-based specification will be internally associated with a default population “pop1” in the standardized specification structure. **(B)** The standardized specification structure and model objects are parsed to generate a single set of equations describing the full model given the separate sets of equations for each object.

Specifiers for the higher-level, more abstract model **specification** structure are grouped into populations (each including a name, size, master equations, optional intrinsic mechanism list, and parameters) and connections between populations (each including a direction, connection mechanism list, and parameters) (Figure 7A). Connectivity between populations is specified using connectivity matrices defined in connection mechanisms between presynaptic source and postsynaptic target populations. Models specified by the user with strings are always associated internally with a population (named “pop1” by default). Model specification is divided into populations and connections to facilitate network modeling. A population of multi-compartment neurons can be implemented by specifying different compartments using the compartments field in exactly the same way different populations of point neurons are specified using the populations field. Two compartments of the same neuron can be connected by specifying connections, for instance, using the ohmic axial current mechanism (iCOM in Table 1) from the DynaSim library (see DynaSim demos for examples with explicit compartmental dimensions); other forms of inter-compartmental connectivity can be implemented using custom connection mechanisms. More details on modeling multicompartment neurons can be found in the online tutorials.

The lower-level, more detailed **model** definition structure includes a single set of model elements: parameters (scalars, strings), fixed variables (matrices and scalar expressions), functions (of time and state variables), and ODEs/ICs describing system dynamics (i.e., the evolution of state variables over time) (Figure 7B). It is derived automatically from the DynaSim specification and all associated model objects. Model elements are always assigned unique names in the lower-level model structure by adding an object-specific namespace identifier (e.g., “pop1_” for population object “pop1”; “pop1_Na_” for mechanism object “Na” in population “pop1”) to the reusable names given in the object definition (e.g., “pop1_” for state variable “V” in population “pop1”; “pop1_Na_m” for state variable “m” in mechanism “Na”). The same unique state variable and function names are used in the output **data** structure storing the results of simulation.

#### 3.1.3. Linking equations across model objects

Once namespace identifiers are used to assign unique names to all parameters, variables, and functions, then the equations from lower-level mechanisms need to be combined with the equations from higher-level populations and other lower-level mechanisms belonging to the same population. ODEs can be directly combined, but something extra is required to indicate how mechanism functions affect the dynamics of population state variables defined outside the mechanism; for instance, how the sodium current “INa,” defined in mechanism “Na,” affects the voltage “V” of population “pop1.” Linking objects can be a difficult concept to grasp at first, but understanding it is not necessary to use DynaSim.

**Figure 8:**
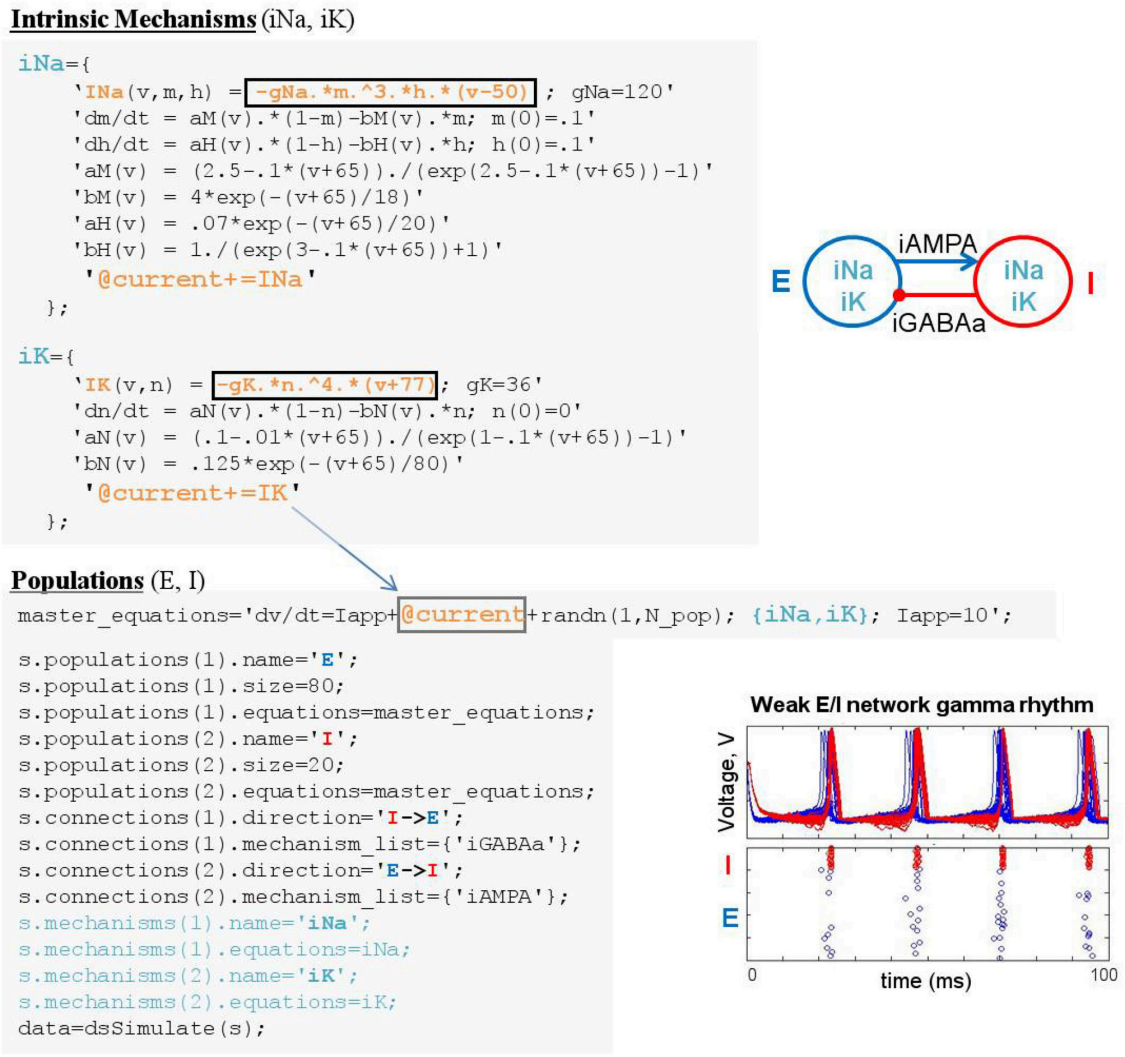
Linking equations across population and mechanism objects. Mechanism linker statements with addition assignment (e.g., @current+=IK) direct DynaSim to substitute functions INa and IK into population-level dynamics “dv/dt,” where the linker appears (i.e., @current). In this example, intrinsic mechanisms are defined in script and added to specification structure in a mechanisms field.

Linking mechanism elements (functions or variables) to equations defined in other objects is achieved by performing substitution guided by “linkers” (Figure 8). A linker is a string that appears in two objects; in one object (e.g., population “pop1”) it is a placeholder indicating the location in an equation (e.g., ODE “dV/dt”) where an element of a different object (e.g., function “INa”) should be inserted; in the second object (e.g., mechanism “Na”) it indicates the element (e.g., function “INa”) to be inserted into the first object. For instance, the linker “@current” can be used in population-level dynamics “dV/dt=@current" along with the mechanism-level linker statement “@current += INa” to direct DynaSim to perform addition assignment, after adding namespace identifiers, resulting in “d(pop1_V)/dt=@current+pop1_Na_INa.” Compound assignment operators (e.g., “+=” and “–=”) enable the same linker to be used in multiple mechanisms; for instance, “@current+=INa” in mechanism “Na” and “@current+=”IK” in mechanism “K” would produce “d(pop1_V)/dt=@current+pop1_Na_INa +pop1_K_IK.” All linkers are removed from the resulting ODE system before simulation; e.g., producing the desired final ODE “d(pop1_V)/dt=pop1_Na_INa+pop1_K_IK.” The online documentation explains how to achieve greater modularization for linking objects with different linker names.

Linking objects is the most unconventional aspect of modeling in DynaSim; it enables the flexible, modular construction of arbitrary dynamical systems, not only neural models. In practice, it is not necessary to understand linkers to build models in DynaSim when working with existing objects from the library. For instance, “@current+=” is used in all prepackaged ionic mechanisms; thus, for conductance-based neural models, users only need to list the ionic mechanisms they wish to include in a population or connection between populations with suitable dynamics. Additional mechanisms can be flexibly added or removed simply by updating the appropriate mechanism list without being concerned with linkers. This frees the modeler to focus on the mechanisms that are most relevant for their models and the parameters of those mechanisms.

#### 3.1.4. Simulation batches

Simulation batches are sets of simulations that systematically vary some aspect of a base model; each simulation in a batch involves some set of modifications to the base model. More precisely, modifications are ways of modifying specifiers (most commonly parameter values) in the base model's high-level specification. Simulation batches are specified using the dsSimulate vary option, which is expanded into a set of modifications for each simulation (see Example 4 for additional details). A “study” in DynaSim is a processing chain that includes a simulation batch plus downstream analysis and visualization.

### 3.2. Simulation

**Figure 9:**
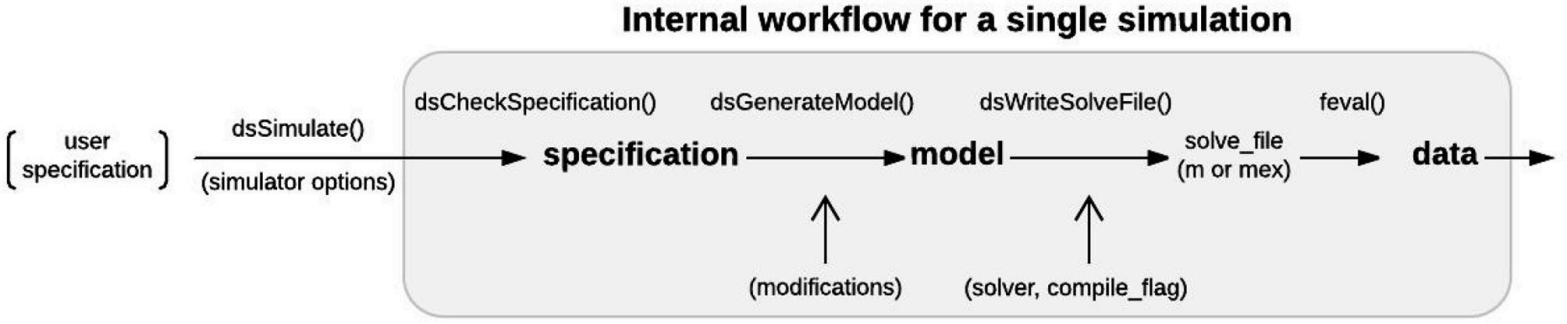
Single simulation workflow. From the user perspective, the functional interface to DynaSim involves specifying a model using strings or a DynaSim specification structure, passing it to dsSimulate, and obtaining a DynaSim data structure with the results of simulation. Internally, dsSimulate standardizes the supplied specification using the dsCheckSpecification function. The standardized specification structure is converted into a DynaSim model structure (Figure 7) using the dsGenerateModel function, which adds object-specific namespace identifiers and links variables and functions across model objects (Figure 8). A solve_file for numerical integration is automatically generated from the model structure by dsGetSolveFile according to simulator options. Simulated data is then obtained by evaluating the solve_file. DynaSim structures are shown in bold. Functions are followed by “().” Simulator options are enclosed in parentheses.

Models are simulated in DynaSim by passing the user's model specification to the dsSimulate function along with options specifying details of the simulation. dsSimulate provides options to control the solver and machine(s) used for numerical integration, the location of outputs, and the details of batch simulation. Depending on the options specified, dsSimulate automates the construction of the full system of equations, as described above, and the generation of MATLAB functions that perform the numerical integration. DynaSim supports custom fixed-step integration (Euler, 2nd-order Runge-Kutta, and 4th-order Runge-Kutta) as well as MATLAB's built-in variable-step solvers (e.g., ode23, ode45). The integration method is specified by the solver option. When fixed-step simulation is desired, DynaSim generates and executes a standalone m-file that explicitly integrates the system of equations using the desired method. When built-in solvers are used, DynaSim automatically generates an m-file with the appropriate format and passes it as a function handle to the desired built-in MATLAB function; consequently, dsSimulate can serve as a simpler interface for using MATLAB's advanced numerical methods. All m-files generated are saved by default and available for examination and re-use. By default, all time points for all state variables are recorded. The number of time points recorded can be decreased by setting the downsample_factor option to an integer value greater than 1. Functions and spike times can be recorded as well using the monitor keyword, as described in the online “Getting started” tutorial. See Figure 9 for additional details on the internal processing performed by DynaSim during each simulation. DynaSim GUI provides an additional interactive interface for real-time simulation using the Euler method with a model stored in an updatable anonymous function that is evaluated at each time step.

A common criticism of simulating computationally intensive models in MATLAB is the time required for simulation. An important method of increasing the simulation speed is available for users with the MATLAB Coder toolbox. When available, the compile_flag option can be used to instruct dsSimulate to compile the automatically-generated m-file into a mex-file with C code. As discussed in the Benchmarking section below, depending on model details, simulating models using compiled C code can reduce simulation time by a factor of 10x.

### 3.3. Batch management

One advantage of DynaSim over other neural simulators is its extensive support for processing sets of simulations (i.e., simulation batches). In practice, one is often interested in how behavior changes as some aspect of a model is varied. To facilitate model exploration, DynaSim offers (1) a compact specification of the parameter space to explore, (2) the ability to perform multiple simulations in parallel on different cores of a single machine (using the Parallel Computing toolbox) and different nodes of a high performance computer cluster (using automated job creation and the qsub command), (3) functions for analysis and visualization of how behavior varies over parameter space, and (4) automated management of large sets of simulation results. See online documentation and Example 6 for details.

### 3.4. Benchmarks

We implemented, adapted, and ran benchmarks taken from a review of simulator tools (Brette et al., 2007). We compared DynaSim with and without C compilation to the Brian 2 simulator with and without C++ compilation (Stimberg et al., 2014). We built our code from the original codebase for the review, available in ModelDB (McDougal et al., 2017) at http://modeldb.yale.edu/83319, including adapting Brian version 1 code for Brian 2. Both our benchmark code and data are available online on GitHub at http://github.com/asoplata/dynasim-benchmark-brette-2007. Two neuron model types were considered: one using integrate-and-fire (IF) type neurons (CUBA) and one using Hodgkin-Huxley (HH) type neurons (COBAHH). Network size was varied across simulations to include 2^0^, 2^1^, …, 2^7^, 250 × 2^0^, 250 × 2^1^, …, 250 × 2^7^ cells. In all cases, 0.5 s of model dynamics was simulated.

**Figure 10:**
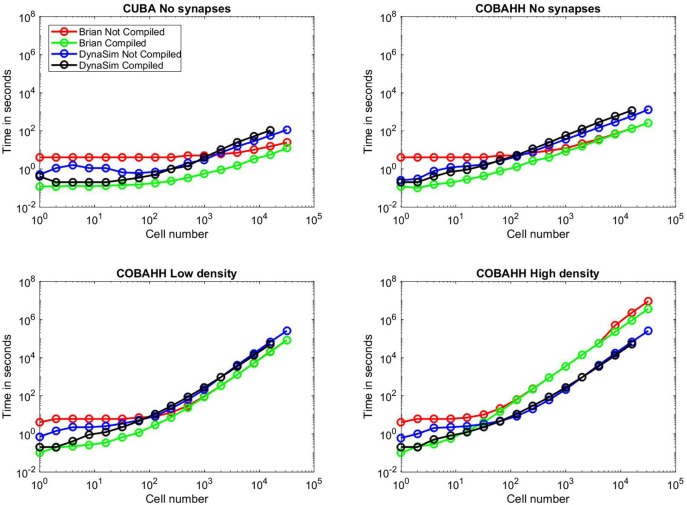
Benchmarks. Time to simulate vs. network size for all benchmarks run; network sizes were 1, 2, 4, 8, 16, 32, 64, 128, 250, 500, 1,000, 2,000, 4,000, 8,000, 16,000, or 32,000 cells. Red lines indicate uncompiled Brian 2 simulation time for given network type and size, green lines indicate time for equivalent C++ compiled Brian 2 simulation, blue lines indicate time for equivalent DynaSim simulation without using MEX compilation, and black lines indicate time for equivalent DynaSim simulation using MEX compilation. **(A)** Benchmarks for simple “current-based” (CUBA) simulations consisting of cells containing just leakage currents and no synapses. **(B)** Benchmarks for Hodgkin-Huxley conductance-based (COBAHH) simulations of cells containing Na, K, and leakage currents and no synapses. **(C)** Benchmarks for COBAHH simulations, but with AMPA and GABA-A synaptic connections at a low density of 2% connection probability. **(D)** Benchmarks for COBAHH simulations, but with AMPA and GABA-A synaptic connections at a high density of 90% connection probability. Note that we could not simulate the highest-sized network (32,000 cells) using compilation under DynaSim, as the resulting data structures were found to be too large to be computed by MATLAB's compiling framework. DynaSim simulations using compilation worked successfully using network sizes of 16,000 cells, and those without compilation could successfully simulate 32,000 cells.

We began by running benchmarks to investigate performance when cells were not connected via synapses, as in Goodman and Brette (2008), in order to evaluate the core simulation speed of intrinsic neuron properties alone. We first ran CUBA simulations (Figure 10A), where cells consist solely of a single leakage current and a thresholded voltage reset. Here, for small networks, both DynaSim and compiled Brian 2 take ~0.1 s to complete the simulation while uncompiled Brian 2 takes ~4 s, owing largely to startup costs. Beyond networks of 1,000 cells, however, DynaSim takes ~50% longer than uncompiled Brian 2 and ~100% longer than compiled Brian 2. Next, we ran COBAHH simulations lacking synapses (Figure 10B). These cells consisted of typical Hodgkin-Huxley sodium, potassium, and leakage currents. As in the CUBA comparison, DynaSim and compiled Brian 2 are faster for smaller networks; however, uncompiled and compiled Brian 2 take less time than DynaSim for networks of more than 100 cells.

Next, we compared the COBAHH model between DynaSim and Brian 2 using synapses with either low (2%) connection densities (Figure 10C) or high (90%) densities (Figure 10D). We used synapses that were more complex than in the original benchmarks (Brette et al., 2007) in two ways: our synapses used “clock-driven,” continuous equations so that they would be updated for all time points instead of just events, and, therefore, the synaptic updates were not uniform, requiring them to be calculated individually. Similarly to the previous synapse-less benchmarks, we chose this method to test the raw synapse simulation speed, since many models require more synaptic history than just the time of a presynaptic event. DynaSim builds a matrix the size of all synapses for all cells and computes the synaptic activity using matrix multiplication at every time step; this implies that DynaSim simulation speed is independent of the connection density. In contrast, Brian 2 uses a synaptic data structure that only contains synapses between neurons with non-zero connection weights. This means that the time taken to simulate a high-density network in Brian 2 can be much longer than that of a low-density network, as one can see when comparing Figure 10C to Figure 10D. With low-density (2%) synaptic connections as in Figure 10C and >100 cells, both uncompiled and compiled Brian 2 perform simulations consistently faster than DynaSim. However, given the same network with a high-density (90%) of synaptic connections in Figure 10D and more than 100 cells, both uncompiled and compiled Brian 2 can take >10x longer than DynaSim. For instance, when running a simulation of 32,000 cells at 90% synaptic connection density, DynaSim took ~3 days to run the simulation, while uncompiled Brian 2 estimated it would take 40 days with comparable “clock-driven" synapses (see below for discussion of “event-driven" synapses), and compiled Brian 2 would still take an estimated 2 weeks.

In every benchmarking scenario, we tested DynaSim with and without C compilation. In all common scenarios, DynaSim with compilation was 1–10x faster than DynaSim without compilation. The benefit of compilation was most significant for networks with fewer than 100 cells, and compiled simulations failed when networks had 32,000 cells (Figure 10). We found an exception for the unusual case of modeling hundreds of completely isolated neurons (i.e., without synaptic connections); understanding the reason for this is complicated by the proprietary nature of the MATLAB Coder. Similarly, Brian 2 compilation was almost always either as fast or faster than uncompiled Brian 2, particularly due to the loss of a startup time. Brian 2 compilation speed converged to the speed of uncompiled simulations, except for the most intensive simulations using large populations connected with a high density, shown in Figure 10D; here, compilation brought back strong gains (30% faster), implying a speedup in total simulation time of weeks. For Brian 2 compilation, we used the GCC compiler with the highly optimizing options “-w,” “-O3,” “-ffast-math,” and “-march=native” since we wanted to push Brian 2 to its speed limits, and could fairly benchmark DynaSim at its fastest against these numbers.

These benchmarks illustrate a frequent answer to the question “which simulator should I use?": it depends. For networks consisting of hundreds of cells, both Brian 2 and DynaSim typically have simulation speeds almost within an order of magnitude of each other. DynaSim excels at larger networks with high synaptic interconnectivity when synaptic dynamics depend on more than spike arrival times (e.g., subthreshold dynamics) and thus require clock-driven computation (i.e., updating synapses at every time step). However, when synaptic dynamics depend only on spike arrival times (i.e., event-driven synapses are acceptable), Brian 2 is able to simulate larger networks orders of magnitude faster (not shown).

### 3.5. Summary of advantages, limitations, and future directions

In this age of incredible computing power, performance is no longer the “one true metric" by which all programs are compared (Rudolph and Destexhe, 2007). With so many competing neural simulators, the choice also depends on ease of use, time to onboard, reproducibility, documentation quality, cross-platform usability, amount of programming knowledge required, etc. Similar to most popular neural simulators, DynaSim supports the Linux, macOS, and Windows operating systems, and it provides the most comprehensive MATLAB-based solution to neural modeling. In contrast, comprehensive python-based solutions have been developed and promoted by NeuralEnsemble (http://neuralensemble.org) and the Human Brain Project (Markram et al., 2011). Compared to many existing neural simulators (e.g., Brian 2, NEURON, NEST, XPP), DynaSim offers better support for batch analysis and visualization (Example 7) and more options for varying model elements across large sets of simulations that can be easily parallelized on multicore processors and computer clusters (Example 6). It also provides a uniquely-powerful graphical interface (Example 8) that enables the exploration of complex neural models by users without programming experience or mathematical expertise. For users with mathematical proficiency, DynaSim offers a “purely” equation-based model specification, similar to Brian 2 and XPP, but lacking in NEURON and NEST. For users desiring to build larger models from existing components, DynaSim offers a modular, object-based specification, similar to Brian 2, NEURON, and NEST, but lacking in XPP. DynaSim also benefits from supporting MATLAB's built-in functions in models and leveraging MATLAB's powerful tools for analyzing and visualizing simulated data; its compatibility with GNU Octave and availability on the Neuroscience Gateway provide many of the same benefits for free to users without a MATLAB license.

At present, DynaSim has several limitations compared to other simulators: (1) it computes synaptic currents at every time step (i.e., synaptic computation is “clock-driven”) rather than only computing when triggered by a synaptic event, (2) it does not manage physical units, thus making users responsible for ensuring consistency, and (3) it does not provide an explicit spatial representation for model objects, although workarounds exist. DynaSim has been tested on MATLAB versions 2013a through 2017b as well as on the latest stable version of GNU Octave (4.2.1). Several features are not currently supported by GNU Octave including the DynaSim GUI, MATLAB Coder for MEX compilation, and parallel simulations using parfor. Despite these limitations, we believe DynaSim offers a competitive mixture of both ease of use and simulating power, especially owing to the built-in parallelization capabilities and its user-friendliness for computational neuroscience novices.

Finally, DynaSim is an open-source project with a growing community of active developers. Progress has already been made at adding support for event-driven synapses, wrappers for popular neuroscience analysis and visualization toolboxes in MATLAB (e.g., FieldTrip, Oostenveld et al., 2011, EEGLAB, Delorme and Makeig, 2004, and Chronux, Bokil et al., 2010), data-driven optimization for parameter estimation (e.g., particle filtering, Meng et al., 2014), and code conversion for interoperability with other simulators via NeuroML (Gleeson et al., 2010). For the latest features and documentation on DynaSim, see http://dynasimtoolbox.org.

## Author contributions

JS designed and implemented the core of the DynaSim Toolbox and Graphical User Interface, wrote the paper, and created the online user documentation. AS was the first alpha tester, helped promote the package, ran benchmark simulations, and contributed to the Benchmarks section. SA was the second alpha tester, added MEX compilation via the MATLAB Coder, and maintained GNU Octave compatibility. DS added parallel processing via the MATLAB Parallel Computing Toolbox. ER helped establish a core team of developers and a development workflow with version control. BP-P helped add support for parallel analysis and plotting of large numbers of simulated datasets. NK supervised the project and encouraged laboratory members to implement models in DynaSim. All authors reviewed the paper.

### Conflict of interest statement

The authors declare that the research was conducted in the absence of any commercial or financial relationships that could be construed as a potential conflict of interest. The reviewer MB declared a shared affiliation, with no collaboration, with the authors to the handling Editor.
